# Efficacy and Safety of Acupuncture on Symptomatic Improvement in Primary Sjögren’s Syndrome: A Randomized Controlled Trial

**DOI:** 10.3389/fmed.2022.878218

**Published:** 2022-05-06

**Authors:** Xinyao Zhou, Haodong Xu, Jinzhou Chen, Hengbo Wu, Yi Zhang, Feng Tian, Xiaopo Tang, Huadong Zhang, Lin Ge, Kesong Li, Wen Jiang, Zhishun Liu, Quan Jiang

**Affiliations:** ^1^Department of Rheumatology, Guang’anmen Hospital, China Academy of Chinese Medical Sciences, Beijing, China; ^2^Postgraduate School, China Academy of Chinese Medical Sciences, Beijing, China; ^3^Intensive Care Unit, Meishan Hospital of Traditional Chinese Medicine, Meishan, China; ^4^Postgraduate School, Beijing University of Chinese Medicine, Beijing, China; ^5^College of Basic Medical Science, Zhejiang Chinese Medical University, Hangzhou, China; ^6^Beijing CreateMed Medicine Technology Co., Ltd., Beijing, China; ^7^Department of Acupuncture and Moxibustion, Guang’anmen Hospital, China Academy of Chinese Medical Sciences, Beijing, China

**Keywords:** Sjögren’s syndrome, acupuncture, dryness, fatigue, pain

## Abstract

**Aim:**

We sought to evaluate the efficacy of acupuncture in treating the main symptoms of primary Sjögren’s syndrome, specifically dryness, pain, and fatigue.

**Methods:**

A total of 120 patients with primary Sjögren’s syndrome were randomized in a parallel-group, controlled trial. Participants received acupuncture or sham acupuncture for the first 8 weeks, then were followed for 16 weeks thereafter. The primary outcome was the proportion of participants with a ≥ 30% reduction in ≥ 2 of 3 numeric analog scale scores for dryness, pain, and fatigue. The secondary outcomes included the European League Against Rheumatism (EULAR) Sjögren’s Syndrome Patient-reported Index (ESSPRI); the EULAR Sjögren’s Syndrome Disease Activity Index; the Schirmer test score; unstimulated saliva flow; serum immunoglobulin G, A, and M concentrations; the Medical Outcome Study Short Form 36 score; salivary gland ultrasound imaging; and the Hospital Anxiety and Depression Scale score.

**Results:**

The proportions of patients meeting the primary endpoint were 28.33% (17/60) in the acupuncture group and 31.66% (19/60) in the sham group, without a statistically significant difference (*P* = 0.705). The IgG concentration at week 16 and the homogeneity in ultrasonography of the salivary glands at week 8 showed significant differences between the 2 groups (*P* = 0.0490 and *P* = 0.0334, respectively). No other differences were observed between the 2 groups. ESSPRI and unstimulated saliva flow were improved in both groups compared to baseline, albeit with a significant difference between them.

**Conclusion:**

In patients with primary Sjögren’s syndrome, acupuncture did not satisfactorily improve symptoms compared to placebo. However, interesting discoveries and possible underlying reasons were demonstrated and discussed, which may be useful to studies in the future.

**Clinical Trial Registration:**

[www.ClinicalTrials.gov], identifier [NCT02691377].

## Introduction

Primary Sjögren’s syndrome (pSS) is a systemic autoimmune disease (1) with the main symptoms of dryness, pain, and fatigue (2), which causes physical and psychological discomfort (3) and reduces the quality of life among both patients whose exocrine glands are affected and those with multiorgan dysfunction. Available treatments are only partially effective for symptom improvement. In the relief of dryness, toadstool alkali agonist showed some effect (4) through the stimulation of salivary secretion. However, in the alleviation of pain and fatigue, no sufficient (5) evidence of treatment efficacy exists thus far. Three randomized controlled trials (RCTs) of hydroxychloroquine (6) were conducted, but none of them reported any statistical improvement in those main symptoms, and a number of studies on infliximab and etanercept (7) left uncertain conclusions about symptom melioration.

Acupuncture, a non-drug therapy that originated in China and has spread worldwide, is often used to improve physical symptoms (8), including dryness, pain, and fatigue. It has been applied for the treatment of Sjögren’s syndrome, but the evidence of its effectiveness is inadequate. A systematic review (9) studied the efficacy of acupuncture on xerostomia and salivary production, but it included 10 RCTs of low quality and was therefore inconclusive. Another systematic review (10) suggests that acupuncture is related to better treatment of dry mouth and eyes in pSS. Due to the uncertainty of the effectiveness of acupuncture in patients with pSS, higher-quality RCTs are necessary to study the efficacy and safety of acupuncture in pSS.

Therefore, in this study, we aimed to evaluate the efficacy of acupuncture on the improvement of the main symptoms (dryness, pain, and fatigue) of pSS over an 8-week treatment period and assess the maintenance of effectiveness throughout a 24-week follow-up period.

## Materials and Methods

### Study Design

This randomized, parallel, sham-controlled trial was conducted at Guang’anmen Hospital (GAMH), China Academy of Chinese Medical Sciences, in Beijing, China. The study course per patient was 24 weeks; baseline measurements were taken at week 0, then followed by 8 weeks of treatment with 20 sessions of acupuncture and 16 weeks of follow-up assessments.

### Participants

Patients were included if they were diagnosed as having primary SS according to the American–European Consensus Group (11) classification criteria, had a disease course of < 10 years, and were > 18 years old. Moreover, they either had to have not taken any medicines for pSS, or had to have stopped them for ≥ 4 weeks before recruitment, or had to be taking the same kinds and doses of non-steroidal anti-inflammatory drugs, oral glucocorticoids, cholinergic agonists, artificial eye drops, and herbal decoctions for ≥ 4 weeks before recruitment, or had to be taking the same kinds and doses of immunosuppressants (e.g., *Tripterygium wilfordii*, methotrexate, cyclophosphamide, cyclosporin, azathioprine, and hydroxychloroquine sulfate) for > 6 months before recruitment.

Participants who met one of the following conditions were excluded: had secondary Sjögren’s syndrome; had a serious systemic disorder (e.g., lymphoma; central nervous system, renal, or pulmonary involvement; myositis; or vasculitis) or severe renal or liver failure; had a history of acupuncture treatments within last 20 days; had an acupuncture contraindication (e.g., allergy to metals, skin lesions on relative acupoints, etc.); had participated in any other clinical trials within the last 30 days before recruitment; were planning to be pregnant or were at risk of becoming pregnant (i.e., no contraception use); had a physical or psychological problem that might confound the trial results, interfere with other subjects’ participation, or might make it risky to follow the investigators’ requirements; and were known for or showed persistent drug or alcohol abuse.

### Randomization and Blinding

Participants were allocated to the acupuncture or sham acupuncture (SA) group with equal chance, based on randomization assignments using a sequence of numbers generated using the Statistical Analysis System version 9.2 (SAS Institute, Cary, NC, United States) by the Institute of Basic Research in Clinical Medicine, China Academy of Chinese Medical Sciences. These assignments were sent to a study member, who placed them into sealed, opaque envelopes with dates and signature labels placed over the seals. This study member subsequently did not take part in any patient interviews, acupuncture sessions, data collection, or statistical analysis. Except for the acupuncturist, all relevant parties were blinded.

### Intervention

During weeks 0–8, 120 patients were randomly treated with acupuncture or SA 3 times a week for the first 4 weeks, then 2 times a week for the next 4 weeks. The needles of the 2 study arms were indistinguishable in appearance and feelings brought to patients. The sham needles were 0.30 mm wide and 25 mm long (12) and consisted of an adhesive pad with a blunt tip. The adhesive pad was made of a sterile cylindrical polyethylene foam (10 mm in diameter and 5 mm in height) with adhesive tape at the bottom. In all, the only difference between the normal and sham needles was that the tips of the sham needles did not taper (13). In the acupuncture group, Hwato acupuncture needles (40 × 0.25 mm; Suzhou Medical Appliance Factory, Suzhou, China) were used. The acupoints used in the acupuncture/SA groups were as follows: bilateral Wai guan (SJ5), bilateral Zhaohai (KI6), Cheng-jiang (RN24), Lianquan (RN23), bilateral Tai yang (EX-HN5), bilateral Cuanzhu (BL2), bilateral Sizhukong (SJ23), and bilateral Jiache (ST6). Equal manipulation *via* twirling, lifting, and thrusting motions of all needles was performed in the acupuncture group but not in the sham group. Needles in both arms were kept in place for 30 min each session, with the Deqi sensation present only in the acupuncture group (14). The same qualified and registered clinical acupuncturist was trained to maintain consistent performance in both the acupuncture and SA groups by following the standard operating procedure formula. After the 8-week intervention, patients were followed for another 16 weeks to evaluate the long-term effectiveness of treatment.

### Endpoints

The primary outcome was the proportion of patients with a ≥ 30% improvement in ≥ 2 of 3 numeric analog scale (NAS) scores for dryness, pain, and fatigue after intervention at week 8 compared to baseline. NAS scores (0–10 points, with 0 points for the best, 10 points for the worst) were requested 3 times a week in week 1–4, and 2 times a week in week 5–8, in total of 20 NAS scores in 8-week intervention, and in week 12, 16, 20, and 24 for follow-up with different measurements. The mean scores of NAS from weeks 2 to 8 were set as the primary endpoint, while those of week 0 were considered the baseline NAS scores. The methodology and evidence of setting the requirement of a ≥ 30% improvement in ≥ 2 of 3 indicators is closely related to clinical efficacy and are consistent or similar to the main results of multiple previous clinical trials (5, 8, 15).

The secondary outcomes included NAS scores (mean ± standard deviation [M ± SD]) for dryness, pain, and fatigue as well as the European League Against Rheumatism (EULAR) Sjogren’s syndrome Patient-reported Index (ESSPRI); the EULAR Sjögren’s Syndrome Disease Activity Index (ESSDAI) (16); the Medical Outcome Study Short Form 36 (SF-36) score; the Hospital Anxiety and Depression Scale (HADS) score (17); the Schirmer test score; unstimulated saliva flow; the erythrocyte sedimentation rate (ESR); the C-reactive protein (CRP) level; serum immunoglobulin (Ig) G, IgA, and IgM levels; and an ultrasound examination of the salivary gland structures.

All salivary gland ultrasonography examinations were performed independently by the same examiner with professional training and sufficient operating experience. Homogeneity, the presence of hypoechogenic areas and hyperechogenic reflections, and the clearness of salivary gland borders were scored according to the scoring system proposed by De Vita (18, 19). The scores of these 4 parameters for the 4 salivary glands were then summed up, ranging from 0 to 48 points for each. Scores calculated at weeks 0 and 8, respectively, were compared.

### Statistical Analysis

The sample size was pre-decided by the hypothesized primary outcome. According to an existing literature review (5), the proportion of patients who reported a ≥ 30% improvement in NAS scores of ≥ 2 of the 3 items with placebo treatment was 17.9%. We used this proportion for our placebo group because no data were available for SA at the time. A sample size of 52 patients per group would be sufficient to detect a 2% null difference in proportions, with a 2-sided 5% level of significance and a power of 80%. The number of subjects was increased to 60 participants in each group (for a total of 120 participants) when a dropout rate of 15% was taken into consideration.

Statisticians and investigators were blinded to the patients’ allocation. Statistical analysis was performed using Stata version 15.1 (StataCorp LLC, College Station, TX, United States) and GraphPad Prism version 8.4 (GraphPad Software, San Diego, CA, United States). We used the first ESSPRI and NAS scores as the baseline scores. Thus, participants without a single session of acupuncture were excluded from statistical analysis. Missing data for the primary outcome were extrapolated using the last-observation-carried-forward (LOCF) approach.

Continuous data are presented using M ± SD values if they were normally distributed; otherwise, they are presented as median ± interquartile range values. Categorical data are presented as frequencies and percentages. In comparisons between the 2 groups, the chi-squared test or Fisher’s exact test was used for primary outcomes. For secondary outcomes, a *t*-test was used if the data were normally distributed; otherwise, the Wilcoxon rank-sum test was applied. An analysis of covariance was used for the baseline adjustment analysis of continuous data. For repeated outcome measurements, a general linear model was used for analyzing continuous data.

The threshold of statistical significance of the 2-tailed *P-*value was 0.05. For repeated outcome measurements, the *P*-values were corrected using the Huynh–Feldt adjustment method.

## Results

### Patient Disposition and Demographics

From March 2016 to September 2018, 120 patients with pSS treated at GAMH were recruited into this trial (ClinicalTrials.gov identifier: NCT02691377). The last follow-up assessment of the last patient was completed on March 11, 2019.

**FIGURE 1:**
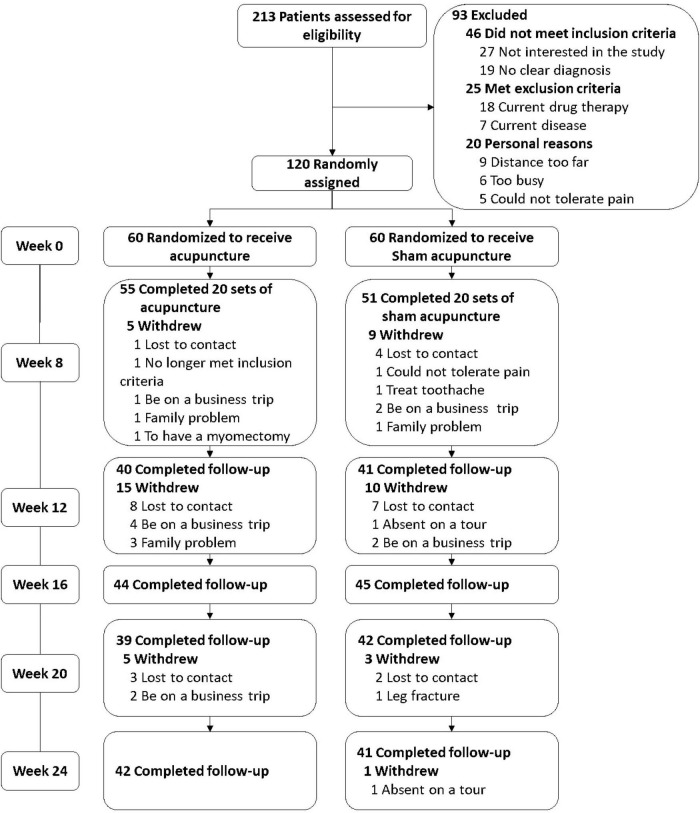
Study flow. The NAS scores at week 0 were was set as baseline of primary outcome. 70.0% (42/60) of the patients received acupuncture between weeks 0 and 8 and then were successfully followed up between weeks 8 and 24, 68.3% (41/60) in the sham group.

All 120 patients were equally (60 patients per group) and randomly assigned to the acupuncture or SA group. The mean age was 56.7 ± 8.2 years in the SA group and 53.8 ± 12.1 years in the acupuncture group, and 88.3% of patients in the SA group and 98.3% of patients in the acupuncture group were female. The disease course of pSS was 35.3 ± 29.4 months in the SA group and 28.9 ± 23.4 months in the acupuncture group, respectively. There were no significant differences in any characteristics between the 2 groups at baseline. Fourteen participants (11.67%) at week 8 and 30 patients (33.3%) at week 24 were lost to follow-up; these losses to follow-up and their reasons are further described in Figure 1. Study participants’ demographic characteristics and concomitant treatment are summarized in Table 1 and their ESSDAI domains are discussed in Table 2.

**TABLE 1:** Characteristics of patients with primary Sjögren syndrome at baseline, by group (*N* = 120).

	Sham acupuncture	Acupuncture	*P*-value
Age, mean (SD), y	56.7 (8.2)	53.8 (12.1)	0.125
Female, No (%)	53 (88.3)	59 (98.3)	0.061
Time to first symptoms, median (SD), m	52.6 (29.8)	46.6 (28.3)	0.260
Time to diagnosis, median (SD), m	35.3 (29.4)	28.9 (23.4)	0.192
Anti-SSA antibodies, No (%)	45 (75.0)	44 (73.3)	0.835
Anti-SSB antibodies, No (%)	27 (45.0)	26 (43.3)	0.854
Abnormal Schirmer test result*^a^* (25th–75th percentile)	2.5 (1.0–5.5)	2.25 (0.75–4.00)	0.983
Decreased unstimulated salivary flow, (25th–75th percentile)	0.45 (0.15–1.02)	0.38 (0.20–1.11)	0.446
Previous systemic involvement,*^b^* No (%)	10 (16.67)	12 (20.00)	0.637
Previous treatment with another immunosuppressant, No (%)	18 (30.00)	23 (38.33)	0.336
Concomitant treatment, No (%)			
Hydroxychloroquine	7 (11.67)	8 (13.33)	0.783
Glucocorticoids	3 (5.00)	2 (3.33)	0.648
Total glucosides of peony	2 (3.33)	4 (6.67)	0.402
Current systemic involvement,*^c^* No (%)	35 (58.33)	34 (56.67)	0.854
ESSDAI*^d^* median (25th–75th percentile)	1.0 (0–2.0)	1.0 (0–2.5)	0.414
ESSPRI*^e^* mean (SD)	5.30 (1.79)	5.03 (1.47)	0.369

*ESSPRI, EULAR Sjögren’s Syndrome Patient-Related Index (the mean of the 3 NAS scores); NAS, numeric analog scale, from 0 (best) to 10 (worst); ESSDAI, EULAR Sjogren’s syndrome disease activity index.*

*^a^Schirmer test ≤ 5 mm in 5 min.*

*^b^Previous systemic involvement is defined in the case report form by history of synovitis; myositis; cutaneous, pulmonary, renal, central nervous system, and peripheral nervous system involvement; or lymphoma.*

*^c^Current systemic involvement is defined in the case report form by presence of synovitis; myositis; or cutaneous, pulmonary, renal, central nervous system, or peripheral nervous system involvement.*

*^d^Ranging from 0 (best) to 123 (worst).*

*^e^Ranging from 0 (best) to 10 (worst).*

*P > 0.05 in all characteristics.*

**TABLE 2:** Patients with active disease, domains of the ESSDAI at enrollment, by Group.

ESSDAI domain*^a^*	No. (%)		*P*-value
	Sham acupuncture (*n* = 54)	Acupuncture (*n* = 56)	
Articular	5 (9.3)	4 (7.1)	0.686
Biological	25 (46.3)	26 (46.4)	0.989
Glandular	4 (7.4)	4 (7.1)	0.957
Pulmonary	2 (3.7)	0 (0.0)	0.239
Hematologic	2 (3.7)	1 (1.8)	0.537
Skin	4 (7.4)	3 (5.4)	0.660
Peripheral neuropathy	1 (1.9)	1 (1.8)	0.979
Constitutional	1 (1.9)	2 (3.6)	0.580
Muscular	1 (1.9)	1 (1.8)	0.979
Central nervous system	1 (1.9)	1 (1.8)	0.979
Lymphadenopathy	4 (7.4)	0 (0.0)	0.055
Renal	2 (3.7)	0 (0.0)	0.239

*^a^Clinical involvement corresponding to each of the 12 domains was defined according to the ESSDAI.*

*P > 0.05 in all items.*

### Primary Outcome

At week 8, the proportions of patients meeting the primary endpoint (≥ 30% reduction in ≥ 2 of the 3 NAS scores for dryness, pain, and fatigue) were 28.33% (17/60) in the acupuncture group and 31.66% (19/60) in the SA group, without a statistically significant difference (*P* = 0.705 after LOCF imputation) (Table 3).

**TABLE 3:** Number (proportion) of patients reaching the primary outcome at different weeks.

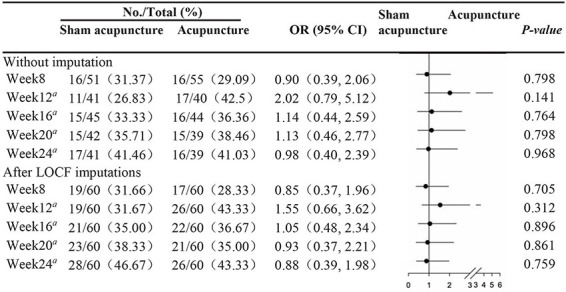

*LOCF, last-observation-carried-forward; OR, odds ratio. Actual values of odds ratio (OR) in this table are plotted on a log scale.*

*^a^Post hoc analysis.*

In addition, we also compared the proportions of patients who experienced a ≥ 30% reduction in 2 of the 3 NAS scores in the 2 groups in other weeks during follow-up (Table 3), including the actual values of the odds ratios. However, no significant difference between the groups was obtained at any week.

### Secondary Outcomes

**FIGURE 2:**
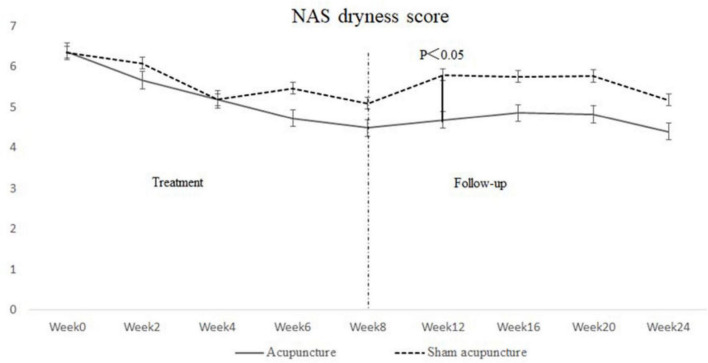
Numeric analog scale (NAS) dryness score at weeks 0, 2, 4, 6, 8, 12, 16, 20, and 24.

No difference in NAS scores for dryness, fatigue, or pain was observed between the 2 groups during any week. However, there was a general decreasing trend in NAS scores observed in both groups during the intervention and follow-up periods (Table 4 and Figure 2).

**TABLE 4:** Numeric analog scale*^a^* scores for dry, pain, and fatigue between weeks 0 and 24, by group.

	Sham acupuncture		Acupuncture		Differences in changes from baseline (Week 0) score adjusted on baseline score, mean (95%)	*P-*value
Domain, by week	NO.	Score, mean (SD)	NO.	Score, mean (SD)		
**Dryness**						
0	60	6.37 (2.20)	60	6.33 (1.84)		
8	52	5.31 (2.11)	55	5.01 (1.94)	−0.22 (−0.86, 0.41)	0.4817
12*^b^*	42	5.79 (2.17)	42	4.68 (2.16)	−0.69 (−1.51, 0.14)	0.1009
16*^b^*	45	5.88 (2.21)	45	4.85 (2.18)	−0.51 (−1.36, 0.34)	0.2393
20*^b^*	45	5.77 (2.23)	39	4.82(2.02)	−0.63 (−1.51, 0.25)	0.1598
24*^b^*	43	5.24 (2.43)	42	4.44 (2.40)	−0.50 (−1.45, 0.45)	0.2980
**Pain**						
0	60	4.09 (2.26)	60	3.75 (2.30)		
8	51	3.46 (2.11)	55	3.15 (1.86)	0.04 (−0.72,0.79)	0.9218
12*^b^*	41	3.67 (2.30)	40	3.21 (1.99)	0.10 (−0.87,1.07)	0.8418
16*^b^*	46	3.23 (2.33)	44	3.25 (2.00)	0.51 (−0.46,1.48)	0.2943
20*^b^*	45	3.30 (2.45)	41	3.07 (2.12)	0.40 (−0.59,1.39)	0.4260
24*^b^*	42	3.22 (2.21)	40	2.79 (2.00)	0.13 (−0.80,1.07)	0.7764
**Fatigue**						
0	60	5.45 (2.38)	60	5.01 (2.28)		
8	52	4.23 (2.25)	55	4.31 (1.94)	0.52 (−0.33, 1.37)	0.2300
12*^b^*	43	4.63 (2.11)	43	4.05 (2.20)	0.26 (−0.76, 1.28)	0.6138
16*^b^*	46	4.52 (2.57)	44	4.00 (2.31)	0.28 (−0.83, 1.38)	0.6166
20*^b^*	42	4.71 (2.23)	39	3.98 (2.25)	0.19 (−0.94, 1.32)	0.7351
24*^b^*	42	4.25 (2.46)	42	3.75 (2.45)	0.28 (−0.89, 1.45)	0.6341

*^a^From 0 (best) to 10 (worst).*

*^b^Post hoc analysis.*

In a *post hoc* analysis, the minimally clinically important improvements (MCIIs) in NAS scores for dryness, pain, and fatigue were calculated as −1, −2, and −3 point(s) on 10-point scales, respectively. There was no significant difference between the 2 groups in the proportion of patients with MCII scores at week 8 in each of the 3 scales or in ≥ 2 of 3 of the scales, nor was there a significant difference in the proportion of patients with an improvement of 1, 2, or 3 point(s) in the NAS scores for pain, fatigue, or dryness or in ≥ 2 of 3 of the scores (Table 5).

**TABLE 5:** Sensitivity of evolution in NAS scores in dry, pain and weak in patients with pSS, between baseline and week 8.

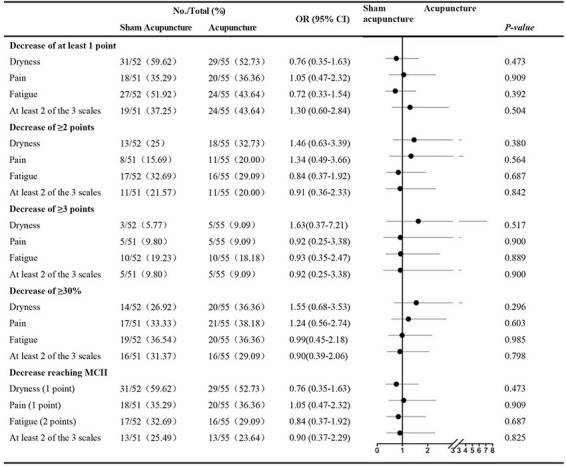

*The NAS scores range from 0 (best) to 10 (worst). MCII indicates minimally clinically important improvement.*

**FIGURE 3:**
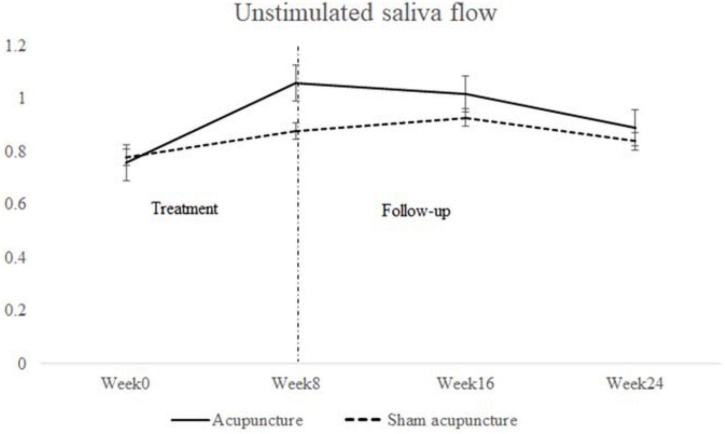
Unstimulated saliva flow at weeks 0, 8, 16, and 24.

In week 16, the IgG concentration (M ± SD) of the SA group showed a significant difference (*P* = 0.0490) compared to that of the acupuncture group (17.65 ± 7.20 g/L vs. 16.97 ± 5.46 g/L) (Table 4). No other difference in ESSPRI, ESSDAI, SF-36, the HADS anxiety subscore, the HADS depression subscore, Schirmer test results, unstimulated saliva flow, ESR, the CRP level, or the IgA or IgM concentration between the 2 groups was obtained in any other week. Compared to week 0, there was a downward trend in unstimulated salivary flow in both groups, more pronounced in the acupuncture group, but no significant difference was found between the two groups (Figure 3). In exploring the differences between before and after the intervention period in the 2 groups, we observed significant differences in ESSPRI between weeks 0 and 8, weeks 0 and 12, weeks 0 and 16, and weeks 0 and 24 between the groups and noted significant differences in unstimulated saliva flow between weeks 0 and 8, weeks 0 and 16, and weeks 0 and 24 between the groups (Table 6).

**TABLE 6:** ESSPRI, ESSDAI, evaluation of disease activity by practitioner, patient-related outcome, and biological variables, by group.

	Sham acupuncture		Acupuncture		Differences in changes from baseline (week 0) score adjusted on baseline score	
Parameter	No.	Value, mean (SD)	No.	Value, mean (SD)	Mean (95% CI)	*P-*value
**ESSPRI*^a^***						
0	60	5.30 (1.79)	60	5.03 (1.47)		
8	51	4.35 (1.95)	55	4.15 (1.50)	0.08 (−0.48, 0.64)	0.7652
12	41	4.75 (1.96)	40	3.95 (1.65)	−0.18 (−0.88, 0.52)	0.6053
16	45	4.60 (2.02)	44	4.02 (1.65)	0.05 (−0.65, 0.75)	0.8767
20	42	4.68 (2.00)	39	4.01 (1.57)	−0.01 (−0.77, 0.76)	0.9881
24	41	4.33 (2.09)	39	3.87 (1.53)	−0.11 (−0.60, 0.82)	0.7546
8–0*^c^*		0.897		0.812		*P* = 0.0001*^d^*/P = 0.0001*^e^*
12–0*^c^*		0.818		1.001		*P* = 0.0002*^d^*/*P* = 0.0016*^e^*
16–0*^c^*		0.922		0. 867		*P* = 0.0001*^d^*/*P* = 0.0032*^e^*
20–0*^c^*		0.875		0.880		*P* = 0.0010*^d^*/*P* = 0.0051*^e^*
24–0*^c^*		1.081		0.969		*P* = 0.0002*^d^*/*P* = 0.0002*^e^*
**ESSDAIs**						
0	54	1.00 (0.0–2.0)	56	1.00 (0.0–2.5)		
8	44	1.0 (0–2.0)	45	1.0 (0–2.0)		
16	44	0.0 (0–1.0)	44	0 (0–2.0)		
24	42	0 (0–2.0)	44	1.0 (0–2.0)		
**SF-36 PH**						
0	54	80.19 (22.96)	56	82.95 (18.94)		
8	48	77.92 (26.95)	52	87.98 (15.28)	−6.40 (−13.76, 0.96)	0.5027
16	45	74.22 (28.18)	43	85.23 (17.83)	−6.09 (−15.09, 2.90)	0.5219
24	43	74.22 (27.32)	40	87.25 (13.54)	−7.18 (−15.50, 1.14)	0.2180
**SF-36 MH**						
0	53	67.08 (19.03)	54	65.28 (17.49)		
8	50	65.50 (20.53)	50	65.00 (19.25)	−0.40(−8.59, 7.79)	0.8266
16	46	67.83 (16.92)	44	67.73 (15.64)	−0.62(−6.85, 5.62)	0.8445
24	45	63.89 (17.55)	44	63.64 (18.41)	−0.07(−6.70, 6.57)	0.6722
**HAD-anxiety**						
0	49	6.27 (4.40)	47	5.47 (3.84)		
8	45	5.58 (3.39)	44	5.52 (4.03)	0.99 (−0.40, 2.37)	0.2132
16	44	5.48 (4.00)	43	5.42 (4.14)	0.89 (−0.50, 2.28)	0.2406
24	43	5.42 (3.90)	40	5.31 (4.07)	1.19 (−0.21, 2.59)	0.1401
**HAD-depression**						
0	49	5.43 (4.11)	47	4.68 (4.11)		
8	45	4.91 (4.12)	44	4.93 (4.05)	0.90 (−0.36, 2.16)	0.1612
16	44	4.98 (4.44)	43	5.40 (5.67)	1.27 (−0.60, 3.13)	0.1932
24	43	5.12 (4.59)	39	5.00 (4.19)	0.92 (−0.58, 2.42)	0.1294
**Schirmer test, mean (SD) median (25th–75th percentile)*^b^***						
0	53	3.42 (2.71) 2.50 (1.00 –5.50)	54	2.88 (2.66) 2.25 (1.00–4.00)		
8	46	3.04 (2.89) 2.00 (1.00–5.00)	50	2.74 (2.12) 2.50 (1.00 –5.00)	0.00 (−0.88, 0.87)	0.8033
16	46	2.75 (2.73) 2.0 (1.0 –4.0)	45	2.87 (2.56) 2.5 (1.0–4.0)	0.22 (−0.72, 1.16)	0.7384
24	43	3.15 (2.55) 2.5 (1.0–5.0)	43	2.85 (2.28) 2.0 (1.5–4.0)	0.20 (−0.84, 1.24)	0.7003
**Unstimulated saliva flow, mean (SD) median (25th–75th percentile)*^b^***						
0	53	0.78 (0.89) 0.47 (0.20–1.02)	55	0.76 (0.86) 0.40 (0.20–1.23)		
8	44	0.88 (0.96) 0.50 (0.30–1.24)	43	1.06 (1.56) 0.51 (0.25–1.19)	−0.15 (−0.54, 0.25)	0.9857
16	46	0.93 (1.00) 0.60 (0.25–1.25)	45	1.02 (1.46) 0.59 (0.30–1.29)	0.00 (−0.41, 0.42)	0.2228
24	43	0.84 (0.73) 0.62 (0.21–1.26)	43	0.89 (0.74) 0.60 (0.34–1.70)	0.00 (−0.31, 0.32)	0.9857
8-0*^c^*		0.10		0.3		*P* < 0.0009*^d^*/*P* = 0.0076*^e^*
16-0*^c^*		0.15		0.26		*P* = 0.0002*^d^ P* = 0.0582*^e^*
24-0*^c^*		0.06		0.13		*P* = 0.0194*^d^*/*P* = 0.0160*^e^*
**ESR, mm/h, mean (SD) median (25th–75th percentile)*^b^***						
0	52	21.67 (14.96) 17.5 (11.5–27.0)	52	22.35 (15.04) 20.0 (12.0–28.50)		
8	44	22.57 (16.45) 19.00 (12.0–26.0)	42	20.67 (15.76) 17.50 (11.00–23.00)	−2.19 (−5.29, 0.91)	0.1941
16	44	20.68 (17.42) 14.50 (8.5–27.0)	43	19.56 (14.46) 16.0 (10.0–23.0)	−0.56 (−4.21, 3.09)	0.6124
24	44	21.09 (17.51) 15.5 (12.0–24.5)	42	20.36 (16.20) 16.50 (11.0–23.0)	−0.78 (−4.85, 3.29)	0.5550
**C-reactive protein, mg/L, mean (SD) median (25th–75th percentile)*^b^***						
0	47	1.71 (3.30) 0.86 (0.35–1.41)	51	1.62 (1.15) 1.24 (0.89–2.26)		
8	40	1.34 (1.24) 1.00 (0.53–1.44)	41	1.89 (2.50) 1.11 (0.49 –2.33)	0.29 (−0.70, 1.28)	0.1447
16	44	1.13 (1.09) 0.89 (0.45–1.19)	42	1.77 (2.12) 1.03 (0.53–2.05)	0.39(−0.46, 1.24)	0.6436
24	43	1.05 (0.92) 0.73 (0.45–1.34)	41	2.72 (4.21) 1.00 (0.42–2.50)	1.32 (−0.15, 2.79)	0.3994
**Serum IgA, g/L**						
0	52	4.58 (5.63)	53	3.00 (1.23)		
8	45	3.53 (3.72)	48	2.63 (1.37)	−0.06 (−0.55, 0.44)	0.2637
16	43	3.80 (4.17)	41	2.94 (1.72)	0.55 (−0.84, 1.95)	0.5905
24	43	2.90 (1.16)	43	2.84 (1.33)	0.82 (−0.39, 2.03)	0.5301
**Serum IgM, g/L**						
0	52	1.67 (2.68)	53	1.18 (0.67)		
8	45	2.09 (4.23)	48	2.83 (5.46)	1.09 (−1.12, 3.30)	0.6107
16	42	1.17 (0.81)	41	1.13 (0.73)	0.67 (−0.27, 1.61)	0.7622
24	42	1.22 (0.88)	39	1.17 (0.74)	0.32 (−0.46, 1.11)	0.5929
**Serum IgG, g/L**						
0	53	18.70 (8.40)	54	18.26 (6.65)		
8	43	18.95 (7.38)	44	18.05 (6.34)	−0.95 (−2.58, 0.67)	0.3016
16	42	17.65 (7.20)	42	16.97 (5.46)	−0.68 (−2.91, 1.55)	0.0490
24	42	17.74 (6.33)	40	17.53 (6.29)	−0.56 (−2.50, 1.37)	0.5265

*ESR, erythrocyte sedimentation rate; ESSDAI, EULAR Sjögren’s Syndrome Disease Activity Index; ESSPRI, EULAR Sjögren’s Syndrome Patient Reported Index; HAD, Hospital Anxiety and Depression scale; SF-36, 36-item Medical Outcomes Study Short-Form Health Survey.*

*^a^The ESSPRI corresponds to the mean of a patient’s numeric analog scale score for dryness, pain, and fatigue.*

*^b^Where the standard deviations exceeded the mean values, medians (25th–75th percentile) are reported as well.*

*^c^Comparisons between different weeks and week 0 in SA and acupuncture group.*

*^d^P in sham group.*

*^e^P in acupuncture group.*

We quantified and calculated the ultrasonography improvement ratio of the 2 groups before and after the 8-week intervention in 4 different dimensions of ultrasonography (18, 19). Both groups acquired improvements in salivary gland ultrasound scores, with a 18.7% improvement in the SA group and a 35.2% improvement in the acupuncture group noted in terms of homogeneity, respectively, and these were significantly different results (*P* = 0.0334). No significant difference was obtained in the other 3 dimensions (Table 7).

**TABLE 7:** Improvement ratio in the ultrasonography of the salivary glands between week 0 and week 8.

	Sham acupuncture improvement ratio %(*N*)	Acupuncture improvement ratio %(*N*)		
No	48	51	*x* ^2^	*P*
Homogeneity	18.7 (9)	35.2 (18)	3.4122	0.0334
Clearness of salivary gland borders	27.0 (13)	27.4 (14)	0.0017	0.1782
The presence of hypoechogenic areals	31.2 (15)	39.2 (20)	0.6865	0.1191
Hyperechogenic reflections	22.9 (11)	25.4 (13)	0.0892	0.1775

*The scoring system by De Vita.*

### Blinding Assessment and Satisfaction

To assess blinding, in week 8, we asked patients if they felt a “deep penetration,” “shallow penetration,” or “no penetration.” Choosing either former two options indicated successful blinding. None of the participants answered “no penetration,” which suggested that the blinding was successful. 78.1% of patients in acupuncture group reported that they had deep penetration, and 60.0% in sham acupuncture group (*P* = 0.122). Additionally, 78.0% of patients were willing to recommend acupuncture treatment to others, showing a good satisfaction.

### Safety

During the first 8 weeks, there was 1 serious adverse event in the placebo group and 2 in the acupuncture group. In the last 16 weeks, there was 1 serious adverse event in the placebo group and none in the acupuncture group. More details of the adverse events are described in Table 8.

**TABLE 8:** Adverse events in patients with primary Sjögren syndrome by group.

Serious adverse event	Sham acupuncture	Acupuncture	Treatment
**Between weeks 0 and 8**			
Upper limb fracture		1	Orthopedic surgery
Dental neuralgia	1		Dental treatment
Uterine fibroids		1	Gynecological operation
Between weeks 8 and 24			
Lower limb fracture	1		Orthopedic surgery
Total events	2	2	

## Discussion

This study did not show that acupuncture improved the main symptoms of pSS compared to placebo treatment, although acupuncture and SA both reduced ESSPRI (M ± SD) and increased unstimulated saliva flow (Table 6). Nearly 33% of participants in both groups (31.66% in the SA group, 28.33% in the acupuncture group) had a ≥ 30% improvement in ≥ 2 of 3 NAS scales at week 8 and met the primary study endpoint. The IgG concentration at week 16 and the homogeneity in ultrasonography of the salivary glands at week 8 showed significant differences between the 2 groups.

The presence of other studies with similar negative outcomes in the literature reflects the persistence of extensive difficulties in alleviating the symptoms of patients with pSS. Hydroxychloroquine, infliximab, etanercept (5–7), and interleukin-6 receptor inhibition (20) did not improve symptoms compared to placebo treatment. The success of B-cell–activating factor receptor blockage for symptomatic improvement was uncertain as well (21).

However, the fact that the IgG concentration differed at week 16 and the homogeneity improved at week 8 in the acupuncture group, with a significant difference compared to the SA group, indicated the existence of an immunity response in pSS patients. The acupuncture regulates the immune system possibly by activating neuronal networks (22) and a variety of bioactive chemicals through peripheral, spinal, and supraspinal mechanisms (23). As a result, the infiltration of lymphocytes into saliva glands might be restrained, and the homogeneity in ultrasound were improved, which was also observed previously by us in a non-obese diabetic mouse model (unpublished data). Further, the symptom of dryness was relieved in both study groups, with a faster decrease in dryness observed in the acupuncture group, but without a statistically significant difference from the SA group. It may be attributed to the small sample size.

In this study, IgG was discovered being different between the 2 groups at week 16, 2 months after the intervention. Previous researches also shared relative conclusions that acupuncture is able to regulate immune systems (24, 25). However, that the difference was observed 2 months after the intervention is in opposition to the stereotype of acupuncture, which typically has strong instant effects (26, 27), and mainly in the area of pain relief. On the contrary, NAS scores for pain in this study did not show improvement, but those for dryness did. The dryness in pSS is led by the injury of salivary glands, which mainly due to immune imbalance. The alleviation of dryness and betterment of IgG observed in this study pointed out that more researches should be launched to unveil the underlying immune mechanism of acupuncture on pSS.

The results that ESSPRI and unstimulated saliva flow were improved significantly in both groups between before and after the intervention, implies that acupuncture benefits patients with symptoms of dryness, though no statistically significant difference was present between the groups. A few other studies have offered similar findings, revealing that acupuncture improved subjective ocular dryness (28) and promoted saliva secretion and symptomatic feelings (29). In terms of fatigue, no sign of fatigue relief was discovered in our study. However, several articles (30–32) mentioned the fatigue relief function of acupuncture or electro-acupuncture in other diseases, probably *via* the regulation of autonomic nerves (33).

The design of the placebo group in this study should be adjusted to avoid a possible efficacy of sham needles, especially in autoimmune diseases, as it was discovered that SA treatment provides some relief from symptoms (34, 35). In our study, the same acupoints in the two groups were stimulated, and the real needles could not penetrate much deeper than the sham ones because most of the acupoints selected were located on bony areas. In other clinical studies, the real needles penetrated much deeper and the sham ones were positioned away from the real acupoints (36, 37). In future studies, real acupoints should be avoided in the sham group, and a better and more specific combination of acupoints is necessary to minimize the efficacy of sham needles.

The real efficacy of acupuncture might not be well demonstrated in this study either, as all participants had consistent acupoints stimulated, which never happens in clinical acupuncture treatment given that acupoints are typically selected according to the individualization principle of traditional Chinese medicine (38). Meanwhile, nearly 33% of our participants met the primary outcome in both groups, indicating that, whether or not the needle penetrates the skin, stimulating acupoints can lead to clinical improvements, but more research is necessary to clarify the nuances of this effect.

## Conclusion

The use of acupuncture did not satisfactorily improve symptoms in patients with pSS compared to those who received placebo treatment. However, interesting discoveries and possible underlying reasons were demonstrated and discussed, which may benefit studies in the future.

## Data Availability Statement

The original contributions presented in the study are included in the article/supplementary material, further inquiries can be directed to the corresponding author/s.

## Ethics Statement

This plan was reviewed and approved by the Ethics Committee of GAMH, China Academy of Chinese Medical Sciences (Ethics number: 2016-005-KY-01), and the study strictly adhered to the spirit of the Declaration of Helsinki of the World Medical Association and the appropriate international ethical guidelines for biomedical research involving humans. All volunteers who entered this clinical trial provided written informed consent.

## Author Contributions

XZ: study design, investigation, data curation, writing—original draft preparation, and project administration. HX: investigation, data curation, partly statistical analysis, writing—review, and editing. JC: study design, manuscript writing, data collection, and submission. HW and YZ: data collection and statistical analysis. KL and WJ: data collection. FT: statistical analysis. XT, HZ, and LG: study conduct and data collection. ZL: conceptualization, methodology, evidence-based acupuncturist, writing—review, and editing, and final improvement of the manuscript. QJ: conceptualization, supervision, and project administration critical revision. All authors read and approved the final manuscript.

## Conflict of Interest

FT was employed by Beijing CreateMed Medicine Technology Co., Ltd., Beijing, China. The remaining authors declare that the research was conducted in the absence of any commercial or financial relationships that could be construed as a potential conflict of interest.

## Publisher’s Note

All claims expressed in this article are solely those of the authors and do not necessarily represent those of their affiliated organizations, or those of the publisher, the editors and the reviewers. Any product that may be evaluated in this article, or claim that may be made by its manufacturer, is not guaranteed or endorsed by the publisher.
